# Nlrp3 Inflammasome Signaling Regulates the Homing and Engraftment of Hematopoietic Stem Cells (HSPCs) by Enhancing Incorporation of CXCR4 Receptor into Membrane Lipid Rafts

**DOI:** 10.1007/s12015-020-10005-w

**Published:** 2020-07-13

**Authors:** Mateusz Adamiak, Ahmed Abdel-Latif, Kamila Bujko, Arjun Thapa, Krzysztof Anusz, Michał Tracz, Katarzyna Brzezniakiewicz-Janus, Janina Ratajczak, Magda Kucia, Mariusz Z. Ratajczak

**Affiliations:** 1 Stem Cell Institute at James Graham Brown Cancer Center, University of Louisville, 500 S. Floyd Street, Rm. 107, Louisville, KY 40202 USA; 2 Center for Preclinical Studies and Technology, Department of Regenerative Medicine at, Medical University of Warsaw, Warsaw, Poland; 3 Division of Cardiovascular Medicine, Gill Heart Institute, University of Kentucky, Lexington, USA; 4 Institute of Veterinary Medicine, Department of Food Hygiene and Public Health Protection, Warsaw University of Life Sciences (WULS-SGGW), Warsaw, Poland; 5 Department of Hematology, University of Zielona Gora, Hospital Gorzow Wlkp, Zielona Góra, Poland

**Keywords:** Nlrp3 inflammasome, Purinergic signaling, Extracellular nucleotides, Complement cascade, Stem cell homing, Stem cell engraftment, Bone marrow sterile inflammation

## Abstract

Fast and efficient homing and engraftment of hematopoietic stem progenitor cells (HSPCs) is crucial for positive clinical outcomes from transplantation. We found that this process depends on activation of the Nlrp3 inflammasome, both in the HSPCs to be transplanted and in the cells in the recipient bone marrow (BM) microenvironment. For the first time we provide evidence that functional deficiency in the Nlrp3 inflammasome in transplanted cells or in the host microenvironment leads to defective homing and engraftment. At the molecular level, functional deficiency of the Nlrp3 inflammasome in HSPCs leads to their defective migration in response to the major BM homing chemoattractant stromal-derived factor 1 (SDF-1) and to other supportive chemoattractants, including sphingosine-1-phosphate (S1P) and extracellular adenosine triphosphate (eATP). We report that activation of the Nlrp3 inflammasome increases autocrine release of eATP, which promotes incorporation of the CXCR4 receptor into membrane lipid rafts at the leading surface of migrating cells. On the other hand, a lack of Nlrp3 inflammasome expression in BM conditioned for transplantation leads to a decrease in expression of SDF-1 and danger-associated molecular pattern molecules (DAMPs), which are responsible for activation of the complement cascade (ComC), which in turn facilitates the homing and engraftment of HSPCs.

**Electronic supplementary material:**

The online version of this article (10.1007/s12015-020-10005-w) contains supplementary material, which is available to authorized users.

## Introduction

Hematopoietic transplantation is based on intravenous infusion of hematopoietic stem progenitor cells (HSPCs), which, in response to bone marrow (BM)-expressed chemoattractants, migrate and home to BM hematopoietic niches [1–3]. This process is followed by their engraftment and expansion to repopulate recipient myeloablated BM. The most important BM chemoattractant is the α-chemokine stromal-derived factor 1 (SDF-1) [4, 5]. However, its homing properties are supported by the bioactive phosphospingolipid sphingosine-1-phosphate (S1P) and by extracellular adenosine triphosphate (eATP) [6–9]. As we demonstrated in the past, for optimal sensing of SDF-1 gradients and intracellular signaling in migrating HSPCs, CXCR4 has to be incorporated into cell membrane lipid rafts [10, 11].

In our previous work we demonstrated that the Nlrp3 inflammasome plays an important role in pharmacological mobilization and egress of HSPCs from BM into peripheral blood (PB) [12, 13]. Specifically, blockade of the Nlrp3 inflammasome by the small-molecule inhibitor MCC950 led to a decrease in the release of HSPCs from BM into PB [12], and this result was subsequently reproduced in Nlrp3-KO mice. In this current investigation we became interested in the role of the Nlrp3 inflammasome complex in the reverse process, which is homing and engraftment of transplanted HSPCs.

The Nlrp3 inflammasome is located in the cytoplasm in an inactive form [14], and upon activation it becomes a functional multiprotein aggregate, composed of several Nlrp3 complex proteins (speck complexes) containing Nlrp3, ASC, and procaspase 1 [15]. Expression of the Nlrp3 inflammasome has been primarily observed in innate immunity cells, including monocytes, macrophages, granulocytes, and dendritic cells [16, 17]. Subsequently, this protein complex was also found in T and B lymphocytes [18, 19].

Here we report for the first time that the Nlrp3 inflammasome complex is also expressed at the mRNA level in murine HSPCs, and its blockade by the small-molecule inhibitor MCC950 or its functional absence in Nlrp3-KO mice leads to defective migration, homing, and subsequent engraftment in myeloablated hosts. Specifically, after exposure to MCC950 or isolation from Nlrp3-KO mice HSPCs show decreased chemotaxis in response to SDF-1, S1P, and eATP gradients. Moreover, we observed that decreased responsiveness of HSPCs to homing factors in BM is related to defective incorporation of the SDF-1 receptor CXCR4 into membrane lipid rafts. This is because Nlrp3 inflammasome activation in HSPCs leads to autocrine secretion of eATP at the leading surface of migrating cells, which promotes incorporation of CXCR4 into membrane lipid rafts.

This finding again confirms the involvement of innate immunity in the regulation of HSPC migration. Importantly, we provide novel evidence that the Nlrp3 inflammasome not only plays a pivotal role in egress/mobilization of HSPCs from BM into PB [12], but its expression in transplanted HSPCs and in cells in the BM microenvironment conditioned for transplantation is crucial for optimal homing, engraftment, and hematopoietic reconstitution.

## Materials and Methods

### Animals

Pathogen-free, 6–8-week-old female C57BL/6 J wild-type (WT) and B6.129S6-Nlrp3^tm1Bhk^/J (Nlrp3-KO) mice were purchased from the Central Laboratory for Experimental Animals, Medical University of Warsaw or the Jackson Laboratory (Bar Harbor, ME; USA) at least 2 weeks before experiments. Animal studies were approved by the Animal Care and Use Committee of the Warsaw Medical University (Warsaw, Poland) and the University of Louisville (Louisville, KY, USA).

### Murine Bone Marrow-Derived Mononuclear Cells (BMMNCs)

Cells were obtained by flushing experimental mouse tibias and femurs. Red blood cells (RBCs) were removed by lysis in BD Pharm Lyse buffer (BD Biosciences, San Jose, CA, USA), washed, and resuspended in appropriate media [20].

### Isolation of SKL Cells

SKL cells were isolated from the BM of C57BL/6 wild type and Nlrp3-KO mice. Briefly, the BM was flushed from the femurs, and the population of total nucleated cells was obtained after lysis of red blood cells (RBCs) using 1 × BD Pharm Lyse buffer (BD Pharmingen, San Jose, CA, USA). The cells were subsequently stained using the following lineage marker-specific antibodies (all from BD Biosciences): PE–anti-TCR γδ, clone GL3; PE–anti-CD11b, clone M1/70; PE–anti-TCR β-chain, clone H57–597; PE–anti-CD45R/B220, clone RA3-6B2; PE–anti-TER-119/erythroid cells, clone TER-119; PE–anti-Ly-6G and -Ly-6C (Gr1), clone RB6-8C5; PE–Cy5–anti-Ly-6A/E (Sca-1), clone D7; fluorescein isothiocyanate (FITC)–anti-CD117 (c-Kit), clone 2B8 for 30 min in medium containing 2% fetal bovine serum (FBS). The cells were then washed, resuspended in RPMI-1640 medium, and sorted using a Moflo XDP cell sorter (Beckman Coulter, Indianapolis, IN, USA) as populations of SKL (Sca-1^+^c-Kit^+^ Lin^−^) cells.

### Transwell Migration Assay

RPMI-1640 assay medium (650 μl) plus 0.5% BSA, with or without stromal-derived factor 1 (SDF-1, 5 ng/ml), sphingosine-1-phosphate (S1P, 0.1 μM), or adenosine triphosphate (ATP, 0.25 μg/ml), was added to the lower chambers of a Costar Transwell 24-well plate (Corning Costar). Aliquots of experimental mouse BMMNC suspension (1 × 10^6^ cells per 100 μl), without any addition or incubated for 1 h with ^10^Panx (WRQAAFVDSY, 40 μM), scrambled ^10^Panx (^SC^Panx, FSVYWAQADR, 40 μM), or apyrase (2 U/ml), were loaded onto the upper chambers with 5-μm pore filters and then incubated (3 h, 37 °C, 95% humidity, 5% CO_2_). An aliquot of cells from the lower chambers was harvested and resuspended in human methylcellulose base medium (R&D Systems), supplemented with murine GM-CSF (25 ng/ml) and IL-3 (10 ng/ml), for determining the number of CFU-GM colonies. Cultures were incubated for 7 days (37 °C, 95% humidity, and 5% CO_2_), at which time the colonies were counted under an inverted microscope [20–22].

### Short-Term Homing Experiments

Experimental mice (WT, WT treated with MCC950 [7 mg/kg, IP, every second day for 12 days], or Nlrp3-KO) were irradiated with a lethal dose of γ-irradiation (10 Gy). Twenty-four hours later, the animals were transplanted by tail vein injection with 5 × 10^6^ BM cells from Nlrp3-KO mice, untreated WT mice, or WT mice treated for 1 h with ^10^Panx (WRQAAFVDSY, 40 μM) or scrambled ^10^Panx (^SC^Panx (FSVYWAQADR, 40 μM). All cells were labeled with PKH67 Green Fluorescent Cell Linker (Sigma-Aldrich, St Louis, MO, USA) according to the manufacturer’s protocol. Twenty-four hours after transplantation, BM cells from the femurs were isolated via Ficoll–Paque and divided into two aliquots. One aliquot of cells was analyzed on a flow cytometer, and the second aliquot was plated in serum-free methylcellulose cultures and stimulated to grow CFU-GM colonies with granulocyte-macrophage colony-stimulating factor (GM-CSF, 25 ng/ml) and interleukin 3 (IL-3, 10 ng/ml). After 7 days of incubation (37 °C, 95% humidity, and 5% CO_2_) the number of colonies was scored under an inverted microscope [8, 21].

### Evaluation of Engraftment

For engraftment experiments, WT mice untreated or treated with MCC950 (7 mg/kg, IP, every second day for 8 days before and 12 days during the experiment) or Nlrp3-KO mice were irradiated with a lethal dose of γ-irradiation (10 Gy). Twenty-four hours later, the animals were transplanted by tail vein injection with 1.5 × 10^5^ BM cells from Nlrp3-KO mice or WT mice untreated or treated for 1 h with ^10^Panx (WRQAAFVDSY, 40 μM) or scrambled ^10^Panx (^SC^Panx (FSVYWAQADR, 40 μM). Twelve days after transplantation, the femora of transplanted mice were flushed with phosphate-buffered saline (PBS). BM cells purified via Ficoll–Paque were plated in serum-free methylcellulose cultures and stimulated for formation of CFU-GM colonies with mGM-CSF (25 ng/ml) and IL-3 (10 ng/ml). After 7 days of incubation (37 °C, 95% humidity, and 5% CO_2_) the number of colonies was scored under an inverted microscope. The spleens were also removed, fixed in Telesyniczky’s solution for CFU-S assays, and the colonies counted on the surface of the spleens [8, 21, 23].

### Recovery of Leukocytes and Platelets

Experimental mice (WT or WT treated with MCC950, 7 mg/kg, IP, every second day for 8 days before and 28 days during the experiment) were irradiated with a lethal dose of γ-irradiation (10 Gy). Twenty-four hours later, the animals were transplanted by tail vein injection with 7.5 × 10^5^ BM cells from Nlrp3-KO mice or WT mice untreated or treated for 1 h with ^10^Panx (WRQAAFVDSY, 40 μM) or scrambled ^10^Panx (^SC^Panx (FSVYWAQADR, 40 μM). Transplanted mice were bled at various intervals from the retro-orbital plexus to obtain samples for white blood cell (WBC) and platelet (PLT) counts, as described [8, 21, 24]. Briefly, 50 μl of PB were drawn into EDTA-coated Microvette tubes (Sarstedt Inc., Newton, NC, USA) and run within 2 h of collection on a HemaVet 950FS hematology analyzer (Drew Scientific Inc., Oxford, CT, USA).

### qRT-PCR Analysis of Nlrp3 Inflammasome Complex Gene Expression

BMMNCs from non-irradiated and irradiated C57BL/6 and Nlrp3-KO mice were isolated, resuspended in RPMI-1640 medium plus 0.5% bovine serum albumin (BSA; Sigma-Aldrich, at 2 million cells per 500 μl of medium), centrifuged, and the total RNA isolated with the RNeasy Mini kit (Qiagen Inc.) after DNase I (Qiagen Inc.) treatment. The purified RNA was reverse-transcribed with MultiScribe reverse transcriptase, oligo(dT), and a random-hexamer primer mix (all from Applied Biosystems Life Technologies, CA, USA). Quantitative evaluation of the target genes was then performed using an ABI Prism 7500 sequence detection system (Applied Biosystems Life Technologies) with Power SYBR Green PCR Master Mix reagent and specific primers. The PCR cycling conditions were 95 °C (15 s), 40 cycles at 95 °C (15 s), and 60 °C (1 min). According to melting point analysis, only one PCR product was amplified under these conditions. The relative quantity of a target gene, normalized to the β2-microglobulin gene as the endogenous control and relative to a calibrator, was expressed as 2^–ΔΔCt^ (fold difference).

The following primer pairs were used for analysis:

*mNLRP1.*

forward primer: 5′- GCT GAA TGA CCT GGG TGA TGG T-3′.

reverse primer: 5′-CTT GGT CAC TGA GAG ATG CCT G-3′.

*mIL-18.*

forward primer: 5′- ACA ACT TTG GCC GAC TTC AC-3′.

reverse primer: 5′-GGG TTC ACT GGC ACT TTG AT-3′.

*mIL-1β.*

forward primer: 5′- TCA CAG CAG CAC ATC AAC AA-3′.

reverse primer: 5′-TGT CCT CAT CCT GGA AGG TC-3′.

*mAIM2.*

forward primer: 5′-AAA ACT GCT CTG CTG CCT CT-3′.

reverse primer: 5′-GAT GGC TTC CTG TTC TGC CA-3′.

*mCasp1.*

forward primer: 5′- CAC AGC TCT GGA GAT GGT GA-3′.

reverse primer: 5′- GGT CCC ACA TAT TCC CTC CT-3′.

*mASC.*

forward primer: 5′- GCC AGA ACA GGC ACT TTG TG-3′.

reverse primer: 5′- AGT CAG CAC ACT GCC ATG C-3′.

*mNlrp3.*

forward primer: 5′- GCT GCT GAA GAT GAC GAG TG-3′.

reverse primer: 5′-TTT CTC GGG CGG GTA ATC TT-3′.

*mHMGB1.*

forward primer: 5′- TAA AAA GCC CAG AGG CAA AA-3′.

reverse primer: 5′- GCS GCS ATG GTC TTC CAC CT-3′.

*mS100A9.*

forward primer: 5′- TGG TGG AAG CAC AGT TGG −3′.

reverse primer: 5′- CAT CAG CAT CAT ACA CTC CTC AA −3′.

*mSDF-1.*

forward primer: 5′- CGT GAG GCC AGG GAA GAG T-3′.

reverse primer: 5′- TGA TGA GCA TGG TGG GTT GA −3′.

*mSCF.*

forward primer: 5′- TAC CAT ATC TCG TAG CCA ACA ATG A − 3′.

reverse primer: 5′- GGC AAA TCF TCC AAA TGA CTA TAT GA −3′.

*mβ2M.*

forward primer: 5′-ATGCTATCCAGAAAACCCCTCAAAT-3.

reverse primer: 5′-AACTGTGTTACGTAGCAGTTCAGTA-3′.

### Lipid Raft Detection

Murine tibias and femurs from C57Bl/6 and Nlrp3-KO animals were flushed, and BM-derived MNCs were obtained after lysis of red blood cells (RBCs) using 1 × BD Pharm Lyse buffer (BD Pharmingen, San Jose, CA, USA). Murine SKL (Sca-1^+^c-Kit^+^Lin^−^) cells were purified by using fluorescence-activated cell sorting (FACS) from BM-derived MNCs using the following lineage marker-specific antibodies (all from BD Biosciences): PE–anti-TCR γδ, clone GL3; PE–anti-CD11b, clone M1/70; PE–anti-TCR β-chain, clone H57–597; PE–anti-CD45R/B220, clone RA3-6B2; PE–anti-TER-119/erythroid cells, clone TER-119; PE–anti-Ly-6G and Ly-6C (Gr1), clone RB6-8C5; PE–Cy5–anti-Ly-6A/E (Sca-1), clone D7; fluorescein isothiocyanate (FITC)–anti-CD117 (c-Kit), clone 2B8. Briefly, SKL cells isolated from Nlrp3-KO and WT BM were plated on fibronectin-coated plates overnight, then incubated with SDF-1 (50 ng/ml) and LL-37 (2.5 μg/ml). SKL cells from WT BM were also exposed to ^10^Panx-1 inhibitory peptide (200 μM; Tocris) or scrambled ^10^Panx peptide (200 μM; Tocris), adenosine (10 μM; Sigma-Aldrich), or CoPP (cobalt III protoporphyrin IX chloride, 50 μM; Enzo Life Science) for 3 h, then washed and fixed in 3.7% paraformaldehyde. The cholera toxin B subunit conjugated with FITC (Sigma-Aldrich) was applied to detect the ganglioside GM1, and rat monoclonal anti-CXCR4 IgG antibody (R&D Systems) and Alexa Fluor 594 goat anti-rat IgG antibody (Invitrogen) were applied to detect CXCR4. The stained cells were examined, and images were generated using a FluoView FV1000 laser-scanning confocal microscope (Olympus America Inc., Center Valley, PA, USA).

### Enzyme-Linked Immunosorbent Assay

Conditioned media (CM) of lethally irradiated (10 Gy of γ-irradiation) WT and Nlrp3-KO mice were prepared as follows. After isolation of whole bone marrow, the cells were incubated in RPMI with 0.5% BSA for 24 h at 37 °C in a 5% CO_2_ incubator. The supernatant (CM) was then harvested. The residual C5a level was measured by enzyme-linked immunosorbent assay (ELISA) according to the manufacturer’s protocols (Abcam, cat. no. ab193718) [25, 26].

### Statistical Analysis

All results are presented as mean ± SD. Statistical analysis of the data was done using Student’s t test for unpaired samples, with *p* ≤ 0.05 considered significant.

## Results

### Normal Murine Hematopoietic Stem/Progenitor Cells (HSPCs) Express Nlrp3 Inflammasome Components

**Fig. 1:**
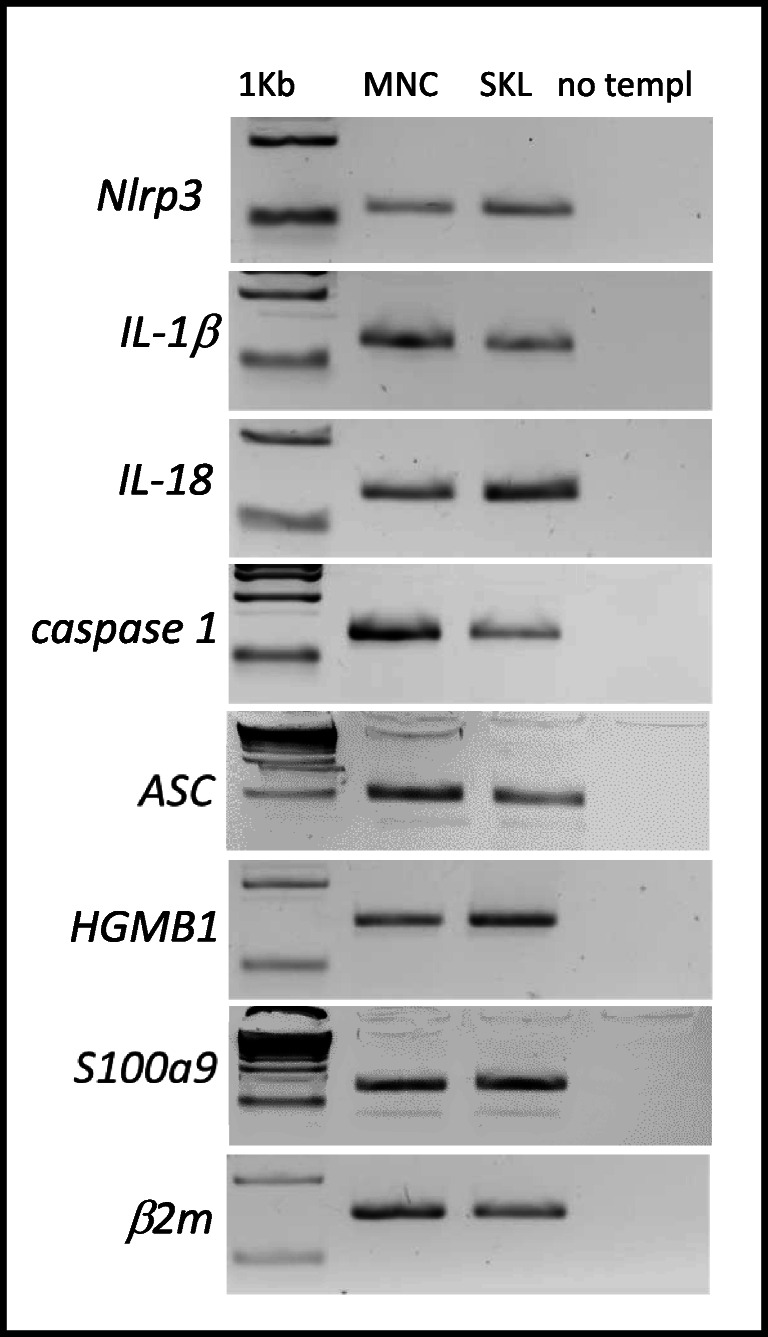
**Expression of Nlrp3, IL-1β, IL-18, caspase 1, ACS, HGMB1, and S100a9 mRNAs in murine BMMNCs and BM-purified SKL cells.** Expression of Nlrp3, IL-1β, IL-18, caspase 1, ACS, HGMB1, and S100a9 mRNAs in BMMNCs and BM-purified SKL cells as measured by RT-PCR. Results are combined from three independent purifications of BM and SKL cells isolated from six animals per purification

The Nlrp3 inflammasome is expressed in innate immunity cells and in cells belonging to the T and B lymphocytic lineages [16–19]. Here we asked whether it is also expressed in murine HSPCs. As shown in Fig. 1**,** all crucial Nlrp3 inflammasome components, including Nlrp3 protein, ASC, caspase 1, IL-1β, and IL-18 are expressed at the mRNA level in murine FACS-purified Sca-1^+^Kit^+^Lin^−^ (SKL) cells. In addition, we also detected HSPC expression of mRNAs for two important danger-associated molecular pattern molecules (DAMPs, also known as alarmines [12]), high mobility group box 1 (HMGB1) protein and the S100 calcium-binding protein S100a9. Both of these DAMPs are released from cells after activation of the Nlrp3 inflammasome.

### Proper Expression of the Nlrp3 Inflammasome in HSPCs Is Required for their Migration toward BM-Homing Chemoattractants

**Fig. 2:**
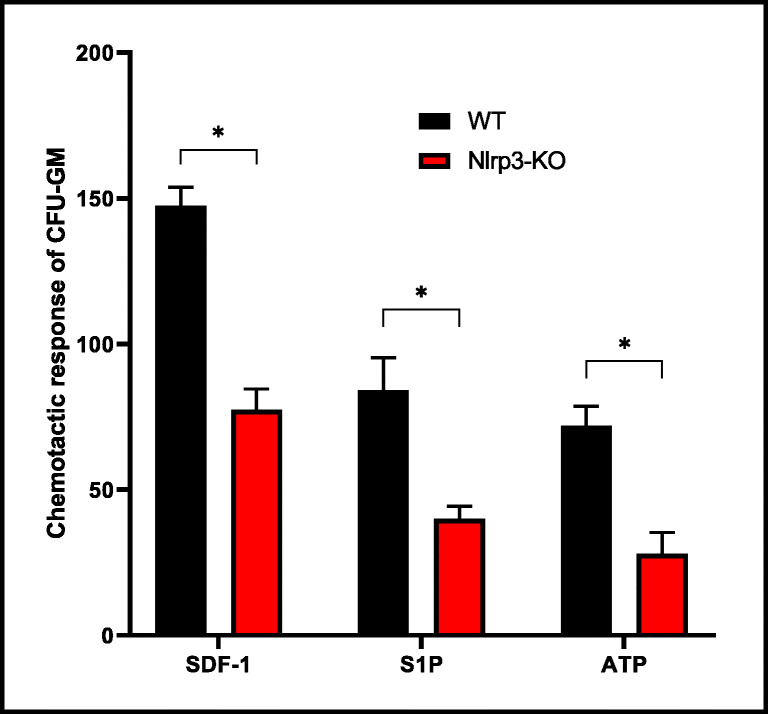
**Impact of Nlrp3 on the chemotactic activity of murine BMMNCs.** The chemotactic responsiveness of BMMNCs from WT or Nlrp3-KO mice to medium supplemented with SDF-1, S1P, or ATP according to the number of CFU-GM clonogenic progenitors. Results are combined from two independent experiments. *p > 0.05

In our previous work we demonstrated that inhibition of Nlrp3 inflammasome expression in mice by its specific inhibitor MCC950 significantly decreases egress of HSPCs from BM into PB during pharmacological mobilization by granulocyte colony-stimulating factor (G-CSF) or the CXCR4 receptor antagonist AMD3100 [12]. We have proposed that this could be a result of decreased migration of cells, and to better address this issue we performed Transwell chemotaxis assays using normal BMMNCs exposed to nontoxic doses of MCC950 and tested against the chemoattractants SDF-1, S1P, and eATP. We found that migration of BMMNCs and CFU-GM clonogenic progenitors was significantly inhibited when Nlrp3 expression in migrating BM cells had been inhibited by nontoxic doses of MCC950 (Supplementary Fig. 1A, B). Next, we reproduced these experiments comparing BMMNCs from control and Nlrp3-KO mice and obtained similar results (Fig. 2). We conclude that proper Nlrp3 inflammasome expression in HSPCs is required for migration of these cells in response to BM-expressed homing factors.

### BMMNCs from Nlrp3-KO Mice Show Impaired Homing and Engraftment after Transplantation into Normal Mice

To address directly the role of the Nlrp3 inflammasome in the homing and subsequent engraftment of HSPCs after transplantation into lethally irradiated mice, we inhibited expression of the Nlrp3 inflammasome in BMMNCs present in the graft by employing the small-molecule inhibitor MCC950 (Supplementary Fig. 2). After exposure, these cells were subsequently infused intravenously into wild type (WT) control animals. We observed that the number of donor-derived PKH67-labeled BMMNCs and the number of CFU-GM clonogenic progenitors, as enumerated 24 h after transplantation in a similar manner as day-12 colony-forming units in spleen (CFU-S) and day-12 CFU-GM clonogenic progenitors isolated from BM, was reduced when Nlrp3 expression was inhibited. In parallel, we also observed a slowing in the recovery kinetics of leukocytes and blood platelets (Supplementary Fig. 2). This result indicates that HSPC expression of the Nlrp3 inflammasome is crucial for in vivo migration of transplanted HSPCs and their homing and engraftment in BM.

**Fig. 3:**
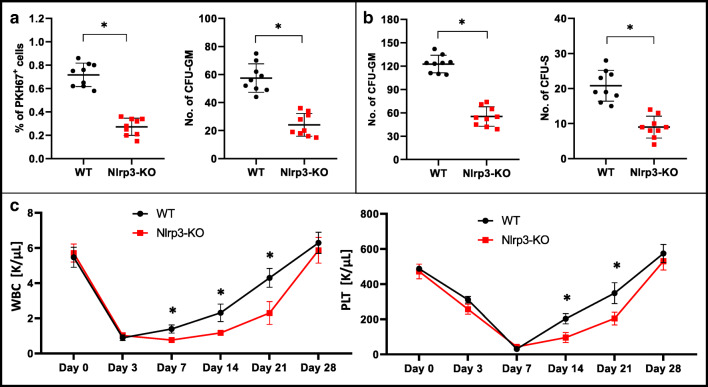
Defect in short- and long-term engraftment of Nlrp3-KO HSPCs in WT mice **Panel A.** Lethally irradiated WT mice (9 per group) were transplanted with bone marrow mononuclear cells (BMMNCs) from WT or Nlrp3-KO mice, which had been previously labeled with a PKH67 cell linker. Twenty-four hours after transplantation, the femoral BMMNCs were harvested, the number of PKH67^+^ cells evaluated by FACS, and the CFU-GM clonogenic progenitors enumerated in an in vitro colony assay. **Panel B.** Lethally irradiated WT mice (9 per group) were transplanted with BMMNCs from WT or Nlrp3-KO mice, and 12 days after transplantation femoral BMMNCs were harvested and plated to count the number of CFU-GM colonies and the spleens removed for counting the number of CFU-S colonies. No colonies were formed in lethally irradiated, untransplanted mice (irradiation control). *p < 0.05. **Panel C.** Lethally irradiated mice (9 per group) were transplanted with BMMNCs from WT or Nlrp3-KO mice. White blood cells (left) and platelets (right) were counted at intervals (at 0, 3, 7, 14, 21, and 28 days after transplantation). *p < 0.05.

To confirm this intriguing observation, we repeated our experiments using BMMNCs from control WT and Nlrp3-KO mice and assayed them in homing and engraftment experiments after transplantation into normal syngeneic WT animals. Figure 3A shows that, compared with WT control cells, the number of fluorochrome-labeled donor cells was reduced by 60% in the BM of transplanted animals 24 h after injection. Similarly, the number of donor CFU-GM in BM after injection of Nlrp3-KO BMMNCs was reduced by more than 50% compared with control WT BMMNCs. In short-term engraftment experiments we observed that, 12 days after transplantation of Nlrp3-KO BMMNCs, the numbers of donor-derived CFU-S progenitors in spleen and CFU-GM progenitors in BM were reduced by more than 50% (Fig. 3B). Corresponding with these results, we observed that mice transplanted with Nlrp3-KO BMMNCs had slower kinetics of recovery for leukocytes and platelets (Fig. 3C).

### Nlrp3 Inflammasome Deficiency in the BM Microenvironment of Transplant-Recipient Mice Also Impairs Homing and Engraftment of BMMNCs

Based on observations that the Nlrp3 inflammasome is upregulated in lethally irradiated mice after conditioning for transplantation, we first exposed recipient mice to MCC950 and transplanted them with syngeneic BMMNCs. Supplementary Fig. 3 shows that the number of donor-derived fluorochrome-labeled cells and CFU-GM clonogenic progenitors was reduced in transplanted mice exposed to MCC950 inhibitor to a similar number as day-12 CFU-S and CFU-GM clonigenic progenitors, compared with transplanted control mice that were not exposed to MCC950. In addition, the recovery of leukocytes and platelet counts was also delayed in mice exposed to an Nlrp3 inflammasome inhibitor around the time of transplantation (Supplementary Fig. 3C**)**.

**Fig. 4:**
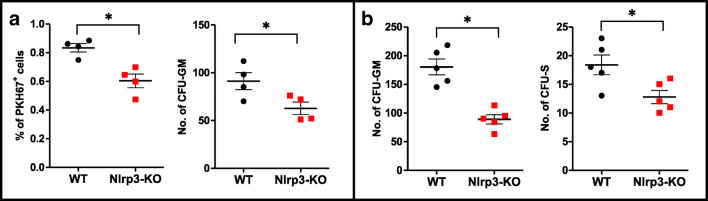
**Defect in homing and short-term engraftment of HSPCs in Nlrp3-KO mice. Panel A.** Lethally irradiated Nlrp3-KO or WT mice (4 mice per group) were transplanted with bone marrow mononuclear cells (BMMNCs) from WT mice, which had been previously labeled with a PKH67 cell linker. Twenty-four hours after transplantation, the femoral BMMNCs were harvested, the number of PKH67^+^ cells evaluated by FACS, and the CFU-GM clonogenic progenitors enumerated in an in vitro colony assay. **Panel B.** Lethally irradiated Nlrp3-KO or WT mice (5 mice per group) were transplanted with BMMNCs from WT mice, and 12 days after transplantation femoral BMMNCs were harvested and plated to count the number of CFU-GM colonies and the spleens removed to count the number of CFU-S colonies. No colonies were formed in lethally irradiated, untransplanted mice (irradiation control). *p < 0.05

Next, we repeated homing and short-term engraftment experiments employing lethally irradiated Nlrp3-KO mice (Fig. 4). Again, as in experiments in which the Nlrp3 inflammasome was inhibited with MCC950, we observed that the number of donor-derived fluorochrome-labeled cells and CFU-GM clonogeneic progenitors was reduced in transplanted Nlrp3-KO mice (Fig. 4A) to a similar number as day-12 CFU-S and CFU-GM clonogenic progenitors (Fig. 4B), compared with control animals.

This result implies that the Nlrp3 inflammasome regulates homing and engraftment, both in transplanted donor-derived HSPCs and in the transplant-recipient BM microenvironment.

### The Nlrp3 Inflammasome Regulates Migration of HSPCs by Promoting Incorporation of CXCR4 into Membrane Lipid Rafts

**Fig. 5:**
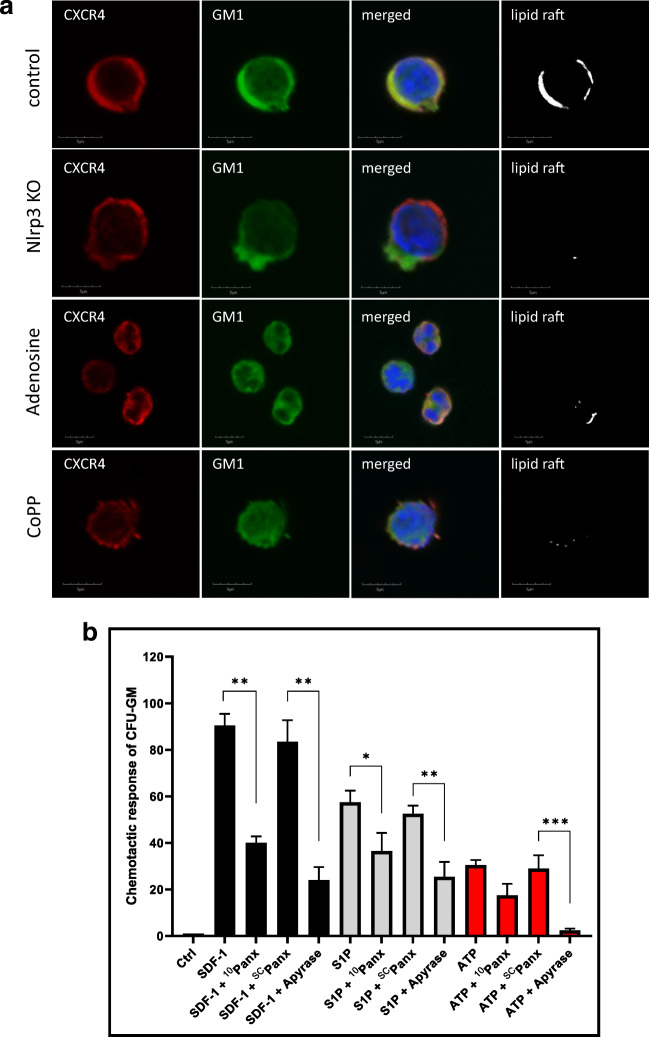
**Confocal analysis of membrane lipid rafts in purified murine SKL cells**. **Panel A.** Defective lipid raft formation in murine C57Bl/6 Nlrp3-KO BM-purified SKL cells or wild type BM-purified SKL cells exposed to adenosine (10 μM) or CoPP (50 μM). Representative images of SKL cells sorted from WT BM, stimulated with SDF-1 (50 ng/ml) and LL-37 (2.5 μg/ml), stained with cholera toxin subunit B (a lipid raft marker) conjugated with FITC and rat anti-mouse CXCR4 followed by anti-rat Alexa Fluor 594, and evaluated by confocal microscopy for formation of membrane lipid rafts. Lipid rafts were formed in SKL cells (control) but not in SKL cells isolated from Nlrp3-KO animals or SKL cells isolated from WT animals after adenosine and CoPP treatment. **Panel B.** The chemotactic responsiveness of mBMMNCs untreated or treated with ^10^Panx, ^SC^Panx, or apyrase in unsupplemented medium or medium supplemented with SDF-1, S1P, or ATP, as determined by counting the number of CFU-GM clonogenic progenitors. Results are combined from two independent experiments. *p > 0.05, **p > 0.01, ***p > 0.001

We reported in the past that migration of HSPCs in response to BM chemoattractants requires incorporation of homing receptors, for example, CXCR4, into cell membrane lipid rafts [10]. Accordingly, certain innate immunity mediators, including cationic antimicrobial peptides (e.g., LL-37, C3a, or β2-defensin) released from BM during myeloablative conditioning for transplantation enhance incorporation of CXCR4 receptor into membrane lipid rafts, and these are required for optimal migration of HSPCs in response to an SDF-1 homing gradient in BM [27]. To address potential involvement of the Nlrp3 inflammasome in this phenomenon, we evaluated membrane lipid raft formation in normal syngeneic BMMNCs and Nlrp3-KO cells in a model in which cells were exposed to a lipid raft-promoting factor, the small immunomodulatory peptide LL-37. Figure 5A shows that, in response to LL-37, CXCR4 became incorporated into membrane lipid rafts in control syngeneic BM-purified SKL cells but not in SKL cells purified from Nlrp3-KO mice (Fig. 5B**)**.

Moreover, in our previous work we demonstrated that the eATP metabolite extracellular adenosine (eAdo) inhibits migration of HSPCs by upregulation of intracellular heme oxygenase 1 (HO-1) in the cells [28]. Based on this finding, during LL-37 priming we exposed murine SKL cells to eAdo (Fig. 5C) or a small-molecule activator of intracellular HO-1, CoPP (Fig. 5D), and observed inhibition of membrane lipid raft formation. This may at least partially explain the negative effect of the eAdo–HO-1 axis on the migration of HSPCs and identifies this axis as a negative regulator of lipid raft assembly.

### Pannexin 1 Channel-Dependent Autocrine Secretion of eATP by HSPCs Promotes their Migration toward Increasing Concentrations of BM-Expressed Chemoattractants

It has been demonstrated that migration of leukocytes in response to a C5a anaphylatoxin gradient is enhanced by autocrine-secreted eATP from migrating cells released on their leading surface [29, 30]. Therefore, we asked whether a similar mechanism occurs for HSPCs migrating in response to SDF-1, S1P, or eATP gradients. We perturbed autocrine secretion of ATP by employing two strategies. First, we inhibited the pannexin 1 channel on the surface of migrating cells by employing a small-peptide inhibitor of the pannexin 1 channel, ^10^Panx, and as a second strategy we exposed migrating cells to apyrase, an enzyme that degrades eATP secreted from the cells. As shown in Fig. 5B**,** chemotaxis of CFU-GM clonogenic progenitors was significantly inhibited in the presence of a pannexin 1 channel inhibitor or in the presence of apyrase.

**Fig. 6:**
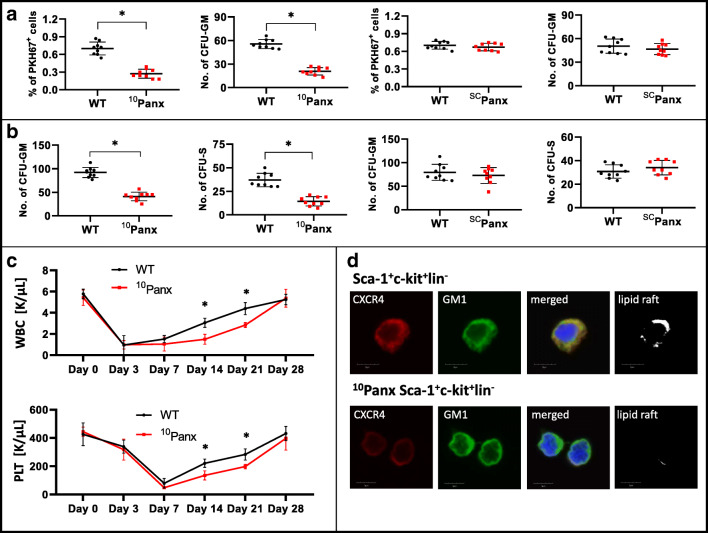
**The impact of pannexin 1 channel blockade on short- and long-term engraftment of HSPCs in WT mice. Panel A.** Lethally irradiated WT mice (9 per group) were transplanted with bone marrow mononuclear cells (BMMNCs) that had been previously labeled with a PKH67 cell linker and then treated with ^10^Panx or ^SC^Panx. Twenty-four hours after transplantation, the femoral BMMNCs were harvested, the number of PKH67^+^ cells evaluated by FACS, and the CFU-GM clonogenic progenitors enumerated in an in vitro colony assay. **Panel B.** Lethally irradiated WT mice (9 per group) were transplanted with BMMNCs treated with ^10^Panx or ^SC^Panx, and 12 days after transplantation the femoral BMMNCs were harvested and plated to count the number of CFU-GM colonies and the spleens removed for counting the number of CFU-S colonies. No colonies were formed in lethally irradiated, untransplanted mice (irradiation control). *p < 0.05, **p < 0.01, ***p < 0.001. **Panel C.** Lethally irradiated WT mice (9 per group) were transplanted with BMMNCs treated with ^10^Panx. White blood cells (above) and platelets (below) were counted at intervals (at 0, 3, 7, 14, 21, and 28 days after transplantation). *p < 0.05. **Panel D**. Defective lipid raft formation in murine BM-purified SKL cells from C57Bl/6 mice exposed to ^10^Panx inhibitory peptide (200 μM). Representative images of SKL cells sorted from WT BM, stimulated with SDF-1 (50 ng/ml) and LL-37 (2.5 μg/ml) (positive control) or exposed to ^10^Panx inhibitory peptide, stained with cholera toxin subunit B (a lipid raft marker) conjugated with FITC and rat anti-mouse CXCR4 followed by anti-rat Alexa Fluor 594, and evaluated by confocal microscopy for formation of membrane lipid rafts. Lipid rafts were formed in SKL cells (control), but not in SKL cells after ^10^Panx inhibitory peptide treatment

These results suggested that eATP, released in an autocrine manner at the cell’s leading surface, promotes incorporation of the CXCR4 receptor into membrane lipid rafts and thus enhances the chemotactic migration of cells. As proof of the autocrine involvement of eATP in this phenomenon, we observed that inhibition of pannexin 1 channels by the small blocking peptide ^10^Panx10, but not by the control scrambled ^SC^Panx peptide, inhibited autocrine release of eATP, which resulted in decreased homing and engraftment of HSPCs. Corroborating this finding, we observed *i)* a reduced number of PKH67^+^-labeled cells and CFU-GM progenitors 24 h after transplantation in recipient BM (Fig. 6A), *ii)* a reduced number of CFU-S colonies in spleens and CFU-GM progenitors in BM 12 days after transplantation (Fig. 6B), and *iii*) impaired recovery of peripheral blood counts in transplanted mice (Fig. 6C). Moreover, we also observed impaired incorporation of CXCR4 into membrane lipid rafts (Fig. 6D).

### Decreased Expression of SDF-1 and Other Factors Involved in Migration and Homing of HSPCs in the BM Microenvironment of Nlrp3-KO Mice Conditioned for Transplantation

**Fig. 7:**
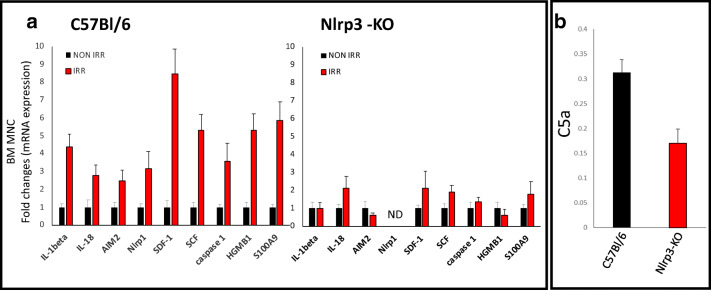
**The effect of myeloablative treatment on mRNA and protein expression related to Nlrp3 inflammasome activation, as measured by qRT-PCR and ELISA. Panel A.** Expression of IL-1β, IL-18, AIM2, Nlrp1, SDF-1, SCG, caspase 1, Hmgb1, and S100a9 mRNAs in bone marrow mononuclear cells (BMMNCs) isolated from non-irradiated and irradiated (1000 cGy) C57Bl/6 and Nlrp3-KO animals, as measured by qRT-PCR. Results of qRT-PCR were normalized to the β2 microglobulin (β2m) level. The data represent the mean value ± SEM for three independent experiments. **Panel B.** The level of C5a protein in conditioned media harvested from irradiated WT or Nlrp3-KO BMMNCs, measured by ELISA. The data represent the mean value for two independent experiments. *p < 0.05

Finally, we became interested in the potential differences in the response of the BM microenvironment to myeloablative treatment in Nlrp3-KO and WT mice. As expected, we observed decreased upregulation of mRNAs for Nlrp3 inflammasome components (ASC, IL-1β, IL-18, and caspase-1) compared with control animals (Fig. 7A). Similarly, the increases in expression of mRNAs for SDF-1 and KL were also at a much lower level in Nlrp3-KO mice than in WT controls. Moreover, we also observed a decrease in expression of mRNA for the Aim2 inflammasome and selected DAMPs, including high mobility group box 1 (HMGB1) protein and the S100 calcium-binding protein S100a9. Of note, all these molecules are important in activating the complement cascade (ComC), which, as we demonstrated in the past, is important for optimal homing and engraftment of transplanted HSPCs [13, 31, 32]. In fact, this trend was paralleled by decreased activation of the ComC in the plasma of Nlrp3-KO mice compared with WT control animals, reflected by a lower level of C5a in an ELISA assay (Fig. 7B).

Finally, it has been reported that the Nlrp3 inflammasome maintains a pool of HSPCs in BM. In support of this finding, Nlrp3 inflammasome-KO mice have a statistically significant reduction in the number of SKL cells, CFU-GM progenitors, and BFU-E progenitors in BM (Supplementary Fig. 4).

## Discussion

The seminal observation of the current report is that the Nlrp3 inflammasome, as expressed in transplanted HSPCs and in the BM microenvironment of recipients conditioned for transplantation, is required for optimal stem cell homing, engraftment, and hematopoietic reconstitution. It has emerged as a major sensor of changes in body microenvironments and is currently the best-studied member of the inflammasome family expressed in hematopoietic and lymphopoietic cells [33, 34], including, as we demonstrate here, normal HSPCs. In fact, hematopoiesis is coregulated by pathways characteristic of the activation of innate immunity cells [35]. This should not be surprising, because of the common developmental origin of these cells from a hemato/lymphopoietic stem cell [36, 37].

After intravenous infusion, HSPCs home from PB to BM stem cell niches in response to chemoattractants secreted in the BM microenvironment, and this process precedes their subsequent engraftment and repopulation of the recipient’s hematopoietic organs [1–3]. It is well known that not all HSPCs in the PB find their way to stem cell niches in the BM, and the majority is trapped in different non-hematopoietic locations in various organs [38]. On the other hand, the fast and efficient homing and engraftment of hematopoietic stem progenitor cells (HSPCs) is crucial for positive clinical outcomes after transplantation. The speed of homing and engraftment can be accelerated by *i)* transplantation of a greater number of HSPCs, *ii)* enhancing their responsiveness to BM-expressed chemoattractants, and, what we also envision, *iii)* enhancing the BM hematopoietic microenvironment of the graft recipient [8, 39–42]. The number of transplanted HSPCs depends on their efficient pharmacological mobilization and harvesting from donor BM and/or their successful ex vivo expansion [42–44].

The process of homing is orchestrated by gradients of factors that induce chemotactic activity in HSPCs, and the list of these chemoattractants is rather short. Specifically, it is well known that, besides SDF-1, HSPCs respond to gradients of S1P, C1P, and eATP [45–47]. The sensitivity of HSPCs to SDF-1 gradients can be enhanced by processing HSPCs for transplantation in hypoxic conditions [49, 50] or exposing them to short-term mild heating (39 °C) [48], short pulses of prostaglandin E2 [51], the inhibitory activity of the SDF-1-degrading enzyme dipeptidylpeptidase 4 (DPP4) [52], or the proper fucosylation of P-selectin glycoprotein ligand 1 on their surface [53].

It is known that the homing receptors are expressed on the surface of cell membranes, which consist of a phospholipid bilayer and several embedded proteins held together via noncovalent interactions between the hydrophobic phospholipid tails. Under physiological conditions, these phospholipid tails are in a liquid crystalline state [11, 54]. Moreover, cell membranes also contain combinations of glycosphingolipids and protein receptors organized into glycoprotein microdomains, known as lipid rafts, and these dynamic microscopic cholesterol-enriched structures are important in assembling signaling molecules together with cell-surface receptors and have been identified as playing a primary role in signaling [55–57].

These lipid rafts play an important role in orchestrating the migration of HSPCs toward higher concentrations of chemotactic factors, and CXCR4, the major homing receptor for SDF-1, is associated with these cell-surface structures [10]. Its presence in cell membranes is required for optimal signaling and chemotactic activity of HSPCs [10]. Several factors have been identified, including anti-microbial cationic peptides, such as *(i)* the complement cascade cleavage fragment C3a, *(ii)* cathelicidin (LL-37) and *iii)* β2-defensin, that are part of the innate immunity response and enhance incorporation of CXCR4 into membrane lipid rafts [10, 26, 46].

**Fig. 8:**
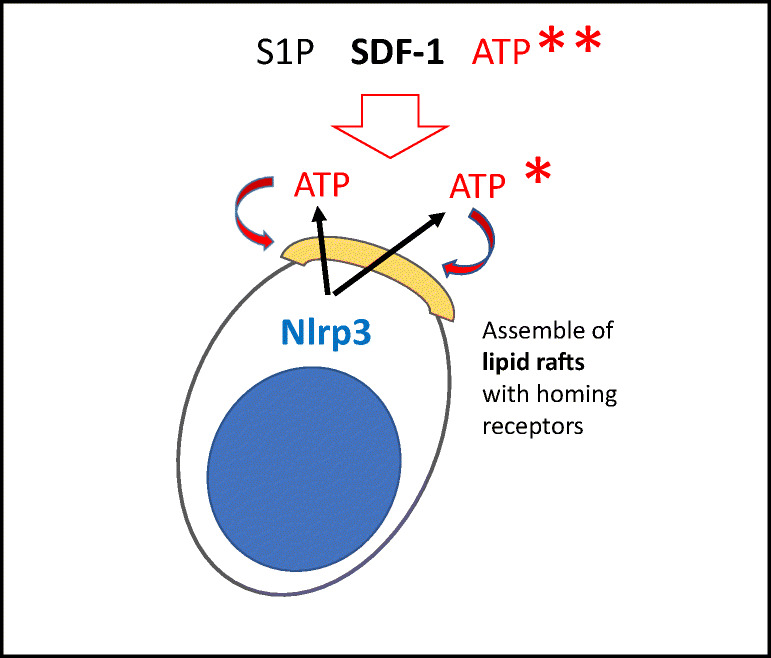
**The role of eATP in the homing and engraftment of HSPCs**. eATP plays a dual role in the homing of HSPCs to BM. On the one hand, whether autocrine-secreted from transplanted HSPCs (*) or secreted in response to conditioning for transplantation from cells in the BM microenvironment (**), eATP promotes formation of membrane lipid rafts (yellow cap) on the surface of HSPCs, which assemble together the major receptors for chemoattractants (SDF-1, S1P, and eATP) (adapted from 65)

We identified a novel mechanism that promotes incorporation of CXCR4 into membrane lipid rafts and depends on activation of the Nlrp3 inflammasome in HSPCs. This activation enhances the release of eATP, which in an autocrine/paracrine manner increases CXCR4 incorporation into membrane lipid rafts at the leading surface of migrating cells and thus facilitates the migration of HSPCs in response to an SDF-1 gradient (Fig. 8). Corroborating such a mechanism, HSPCs isolated from Nlrp3-KO mice or exposed to the eATP-degrading enzyme apyrase have impaired migration toward BM chemoattractants. This result suggests also that a short incubation of HSPCs with eATP before transplantation could improve their BM seeding efficiency, and we are currently testing this possibility.

We hypothesized that exposure of HSPCs to antimicrobial cationic peptides could facilitate lipid raft formation in the mechanism of Nlrp3 inflammasome-mediated autocrine/paracrine release of eATP. In fact, LL-37, which is a very efficient lipid raft formation-promoting peptide [46], failed to induce the formation of membrane lipid rafts in Nlrp3-KO cells, which is similar to the the effect of inhibition of pannexin 1 channel release of eATP. Moreover, we observed that eAdo, a product of eATP metabolism by CD39 and CD73 ectonucleotidases, which inhibits migration of HSPCs [12], in our hands inhibited formation of membrane lipid rafts. Furthermore, experiments are planned to identify which of the P1-binding receptors for eAdo is responsible for this effect. We have also demonstrated that the inhibitory effect of eAdo on migration of HSPCs depends on upregulation of intracellular heme oxygenase 1 (HO-1) [28]. Supporting this observation, we have shown in our current work that upregulation of HO-1 in HSPCs by the small-molecule activator CoPP results in a decrease in membrane lipid raft formation.

The sensitizing effect of autocrine/paracrine-secreted eATP in response to chemoattractants has been reported in an elegant paper that demonstrated a positive effect of eATP and a negative effect of eAdo on the migration of neutrophils in response to a C5a gradient [29]. In our work we report similar results with HSPC responsiveness to BM chemoattractants and provide a molecular explanation of this phenomenon, involving the opposing effects of eATP and eAdo on membrane lipid raft formation.

In addition to modulating the migratory responsiveness of HSPCs to chemoattractant gradients, another important strategy to enhance homing and engraftment of HSPCs is to modify the recipient BM microenvironment in a way that promotes better homing and engraftment of HSPCs. In our previous work we demonstrated that innate immunity and ComC activation play important roles in optimal engraftment of transplanted cells [58]. Here, we observed a homing and engraftment defect in Nlrp3-KO recipient mice, and our ELISA results demonstrated that Nlrp3-KO mice have defective activation of the ComC in CM extracts isolated from BM cells conditioned for transplantation. Therefore, we have demonstrated for the first time that a functional deficiency of the Nlrp3-inflammasome in the BM of transplantation recipients has a negative effect on homing and engraftment of transplanted HSPCs. By way of explanation, our results show that the BM from lethally irradiated Nlrp3-KO mice show a decrease in expression of mRNAs for SDF-1, KL, and certain DAMPs responsible for ComC activation [59–62]. Therefore, the Nlrp3 inflammasome plays an important role, both as regulator of HSPC migration toward BM chemoattractants and as responder to myeloablative irradiation of the BM microenvironment, in facilitating efficient seeding of transplanted cells.

Finally, after they lodge in BM niches HSPCs need to expand and establish proper hematopoiesis. Evidence has accumulated that this process is also regulated by the NLrp3 inflammasome [63]. Specifically, eATP-mediated Nlrp3 inflammasome activation may regulate the pool of HSPCs and seems to be an integrator of metabolic activity in promoting HSPC formation and the development of the myeloid and lymphoid lineages in vivo and in vitro [64]. In fact, we showed in this report that Nlrp3-KO mice have reduced numbers of SKL cells as well as CFU-GM and BFU-E clonogenic progenitors in the BM microenvironment. These observations together demonstrate the important modulatory role of the Nlrp3 inflammasome in hematopoietic reconstitution [65].

In conclusion, we have demonstrated for the first time that the Nlrp3 inflammasome plays a crucial role in homing and engraftment of HSPCs. This effect is mediated in an autocrine/paracrine eATP-dependent manner, both in HSPCs and in cells in the BM microenvironment conditioned by irradiation for transplantation. On the basis of this finding, modulation of Nlrp3 inflammasome activity becomes a potential target for therapeutic interventions to improve clinical outcomes from hematopoietic transplantations. For example, there could be exposure of HSPCs in the graft to eATP, inhibition of autocrine eAdo by CD39 or CD73 blocking agents, or exposure of HSPCs to the Nlrp3 inflammasome-activating antibiotic nigericin. On the other hand, nigericin and eAdo inhibitors could also be employed to increase Nlrp3 expression in the BM of transplantation recipients.

### Grant Information

This work was supported by NIH grants 2R01 DK074720 and R01HL112788, the Stella and Henry Endowment, and the OPUS grant DEC-2016/23/B/NZ3/03157 to MZR. AT was supported by NIH T32 HL134644 Training Grant to MZR. AAL is supported by the University of Kentucky COBRE Early Career Program (P20 GM103527) and the NIH Grant R01 HL124266.

## Electronic supplementary material

Supplementary Figure 1 **Panel A.** Measurement of MCC950 toxicity. Murine BMMNCs were incubated for 1 h with different doses of the Nlrp3-selective inhibitor MCC950, then resuspended in human methylcellulose base medium, supplemented with GM-CSF (25 ng/ml) and IL-3 (10 ng/ml), for determining the number of CFU-GM colonies, and with thrombopoietin (TPO, 100 ng/ml) and IL-3 (10 ng/ml), for determining the number of burst-forming unit-erythroid (BFU-E) colonies. Cultures were incubated for 7 and 14 days respectively (37 °C, 95% humidity, and 5% CO_2_), at which time they were scored under an inverted microscope for the number of colonies. Results from three independent experiments plated in duplicate are pooled together. **Panel B.** The chemotactic responsiveness of mBMMNCs, untreated or treated with MCC950, to medium supplemented with SDF-1, S1P, C1P, or ATP, according to FACS or the number of CFU-GM clonogenic progenitors. Results are combined from two independent experiments. *p > 0.05. (PPTX 65 kb)

Supplementary Figure 2 **Defect in short- and long-term engraftment of HSPCs treated with an Nlrp3-selective inhibitor in WT mice. Panel A.** Lethally irradiated WT mice (9 per group) were transplanted with bone marrow mononuclear cells (BMMNCs) that had been previously treated with MCC950 and labeled with a PKH67 cell linker. Twenty-four hours after transplantation, femoral BMMNCs were harvested, the number of PKH67^+^ cells evaluated by FACS, and the CFU-GM clonogenic progenitors enumerated in an in vitro colony assay. **Panel B.** Lethally irradiated WT mice (9 per group) were transplanted with BMMNCs treated with MCC950, and 12 days after transplantation femoral BMMNCs were harvested and plated to count the number of CFU-GM colonies and the spleens removed for counting the number of CFU-S colonies. No colonies were formed in lethally irradiated, untransplanted mice (irradiation control). *p < 0.05. **Panel C.** Lethally irradiated WT mice (9 per group) were transplanted with BMMNCs treated with MCC950. White blood cells (above) and platelets (below) were counted at intervals (at 0, 3, 7, 14, 21, and 28 days after transplantation). *p < 0.05. (PPTX 74 kb)

Supplementary Figure 3 **Defect in short- and long-term engraftment of HSPCs in WT mice treated with an Nlrp3-selective inhibitor. Panel A.** Lethally irradiated WT mice (9 per group), untreated or treated with MCC950, were transplanted with bone marrow mononuclear cells (BMMNCs) from WT mice that had previously been labeled with a PKH67 cell linker. Twenty-four hours after transplantation, femoral BMMNCs were harvested, the number of PKH67^+^ cells evaluated by FACS, and the CFU-GM clonogenic progenitors enumerated in an in vitro colony assay. **Panel B.** Lethally irradiated WT mice (9 per group), untreated or treated with MCC950, were transplanted with BMMNCs from WT mice, and 12 days after transplantation femoral BMMNCs were harvested and plated to count the number of CFU-GM colonies and the spleens removed for counting the number of CFU-S colonies. No colonies were formed in lethally irradiated, untransplanted mice (irradiation control). *p < 0.05. **Panel C.** Lethally irradiated mice (9 per group), untreated or treated with MCC950, were transplanted with BMMNCs from WT mice. White blood cells (left) and platelets (right) were counted at intervals (at 0, 3, 7, 14, 21, and 28 days after transplantation). *p < 0.05. (PPTX 80 kb)

Supplementary Figure 4 **A reduced number of HSPCs in the BM of Nlrp3-KO mice.** The number of SKL cells in the BM of Nlrp3-KO mice compared with WT control animals was evaluated by FACS and by the number of CFU-GM and BFU-E clonogenic progenitors in in vitro methylcellulose cultures. Results are combined from two independent experiments (4 mice per group per repeat). (PPTX 51 kb)
