# Supplementary figures and images for: Nlrp3 Inflammasome Signaling Regulates the Homing and Engraftment of Hematopoietic Stem Cells (HSPCs) by Enhancing Incorporation of CXCR4 Receptor into Membrane Lipid Rafts
Source: Stem Cell Rev Rep. 2020 Jul 13;16(5):954–67. doi: 10.1007/s12015-020-10005-w (PMC7456406; doi:10.1007/s12015-020-10005-w)

## Slide 1
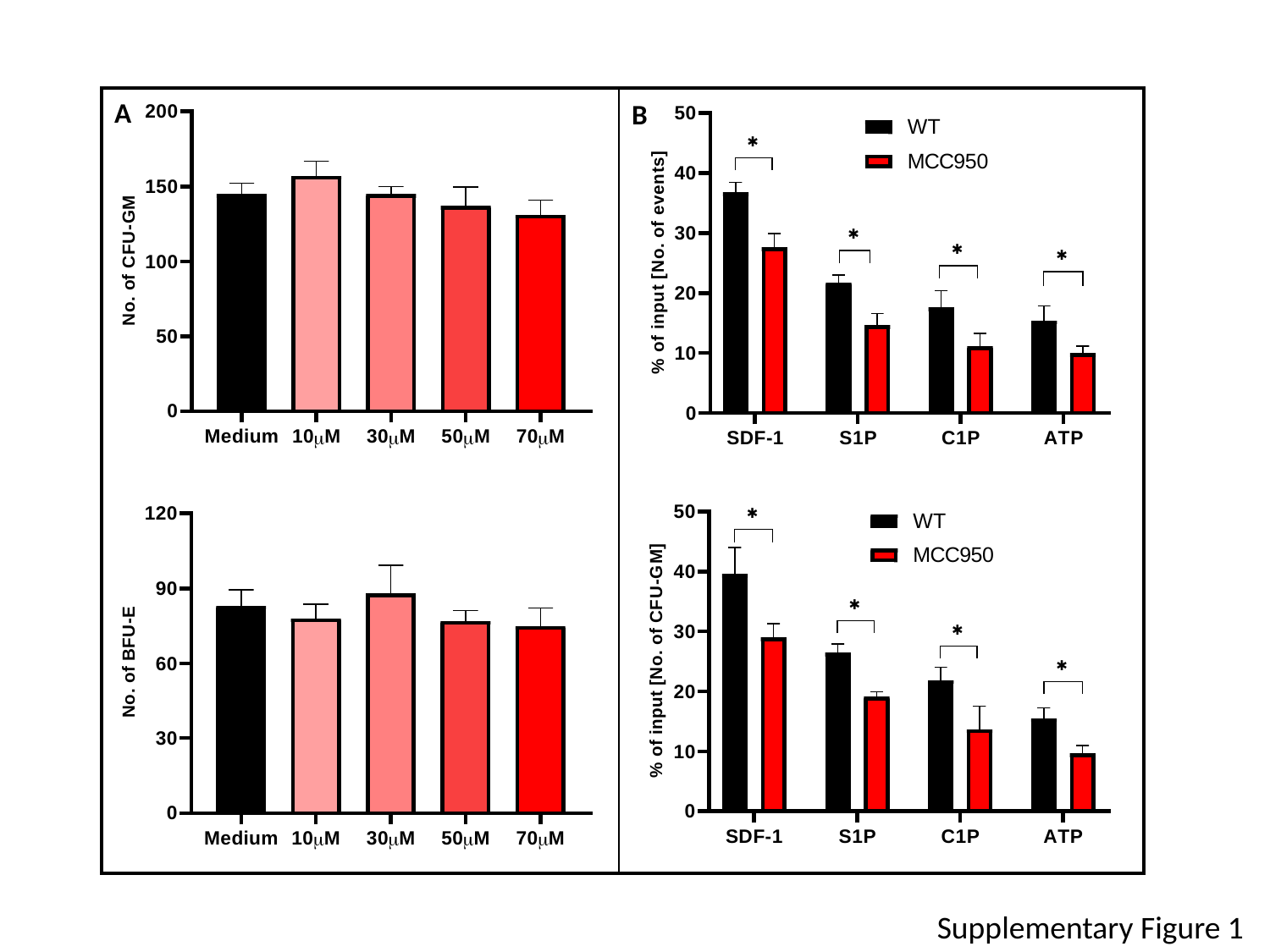

A
B
Supplementary Figure 1

Supplement: Supplementary file 1 — Panel A. Measurement of MCC950 toxicity. Murine BMMNCs were incubated for 1 h with different doses of the Nlrp3-selective inhibitor MCC950, then resuspended in human methylcellulose base medium, supplemented with GM-CSF (25 ng/ml) and IL-3 (10 ng/ml), for determining the number of CFU-GM colonies, and with thrombopoietin (TPO, 100 ng/ml) and IL-3 (10 ng/ml), for determining the number of burst-forming unit-erythroid (BFU-E) colonies. Cultures were incubated for 7 and 14 days respectively (37 °C, 95% humidity, and 5% CO2), at which time they were scored under an inverted microscope for the number of colonies. Results from three independent experiments plated in duplicate are pooled together. Panel B. The chemotactic responsiveness of mBMMNCs, untreated or treated with MCC950, to medium supplemented with SDF-1, S1P, C1P, or ATP, according to FACS or the number of CFU-GM clonogenic progenitors. Results are combined from two independent experiments. *p > 0.05. (PPTX 65 kb) [file 12015_2020_10005_MOESM1_ESM.pptx]

## Slide 1
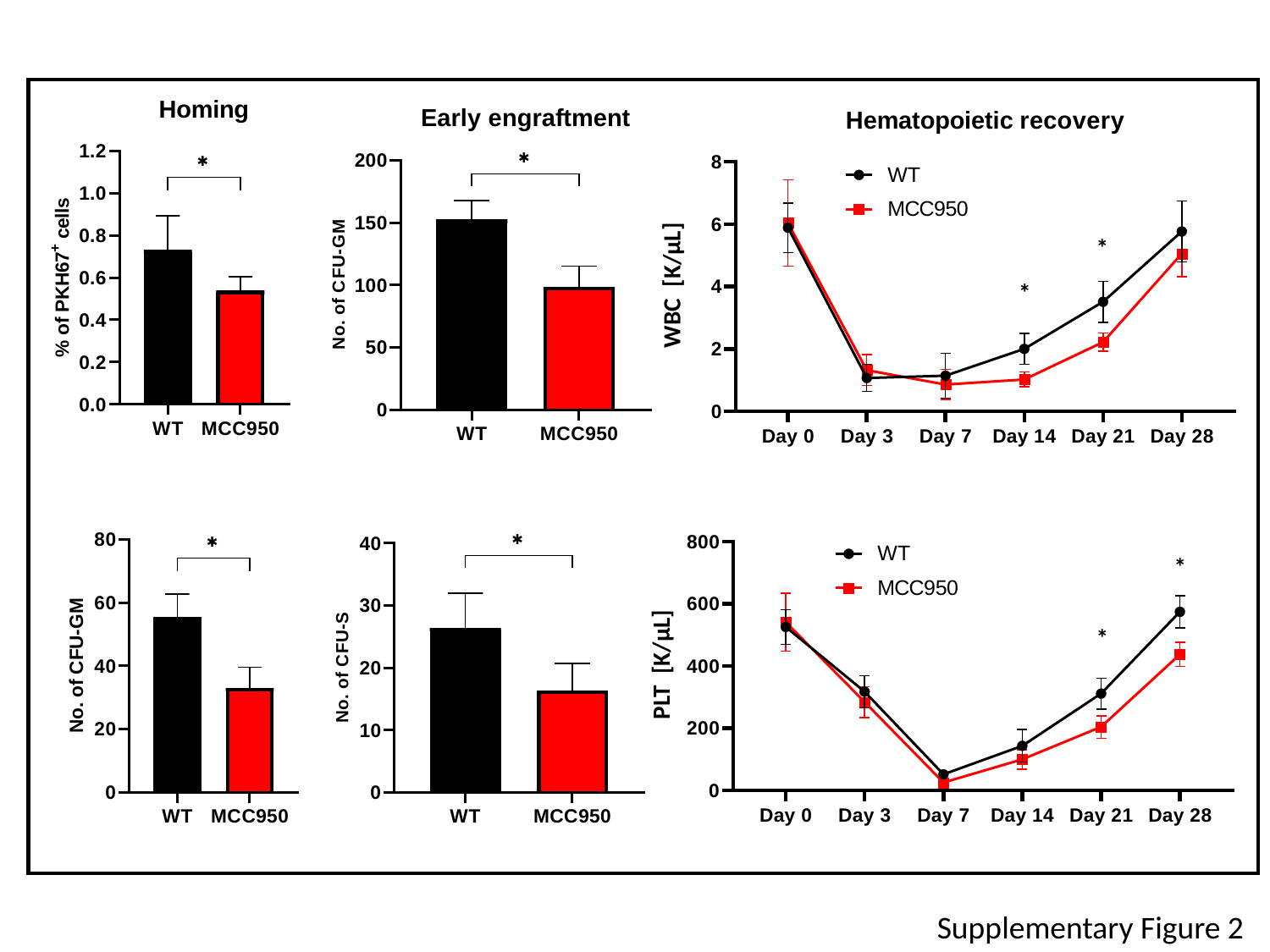

*
*
*
*
WBC [K/µL]
PLT [K/µL]
Supplementary Figure 2

Supplement: Supplementary file 2 — Defect in short- and long-term engraftment of HSPCs treated with an Nlrp3-selective inhibitor in WT mice. Panel A. Lethally irradiated WT mice (9 per group) were transplanted with bone marrow mononuclear cells (BMMNCs) that had been previously treated with MCC950 and labeled with a PKH67 cell linker. Twenty-four hours after transplantation, femoral BMMNCs were harvested, the number of PKH67+ cells evaluated by FACS, and the CFU-GM clonogenic progenitors enumerated in an in vitro colony assay. Panel B. Lethally irradiated WT mice (9 per group) were transplanted with BMMNCs treated with MCC950, and 12 days after transplantation femoral BMMNCs were harvested and plated to count the number of CFU-GM colonies and the spleens removed for counting the number of CFU-S colonies. No colonies were formed in lethally irradiated, untransplanted mice (irradiation control). *p < 0.05. Panel C. Lethally irradiated WT mice (9 per group) were transplanted with BMMNCs treated with MCC950. White blood cells (above) and platelets (below) were counted at intervals (at 0, 3, 7, 14, 21, and 28 days after transplantation). *p < 0.05. (PPTX 74 kb) [file 12015_2020_10005_MOESM2_ESM.pptx]

## Slide 1
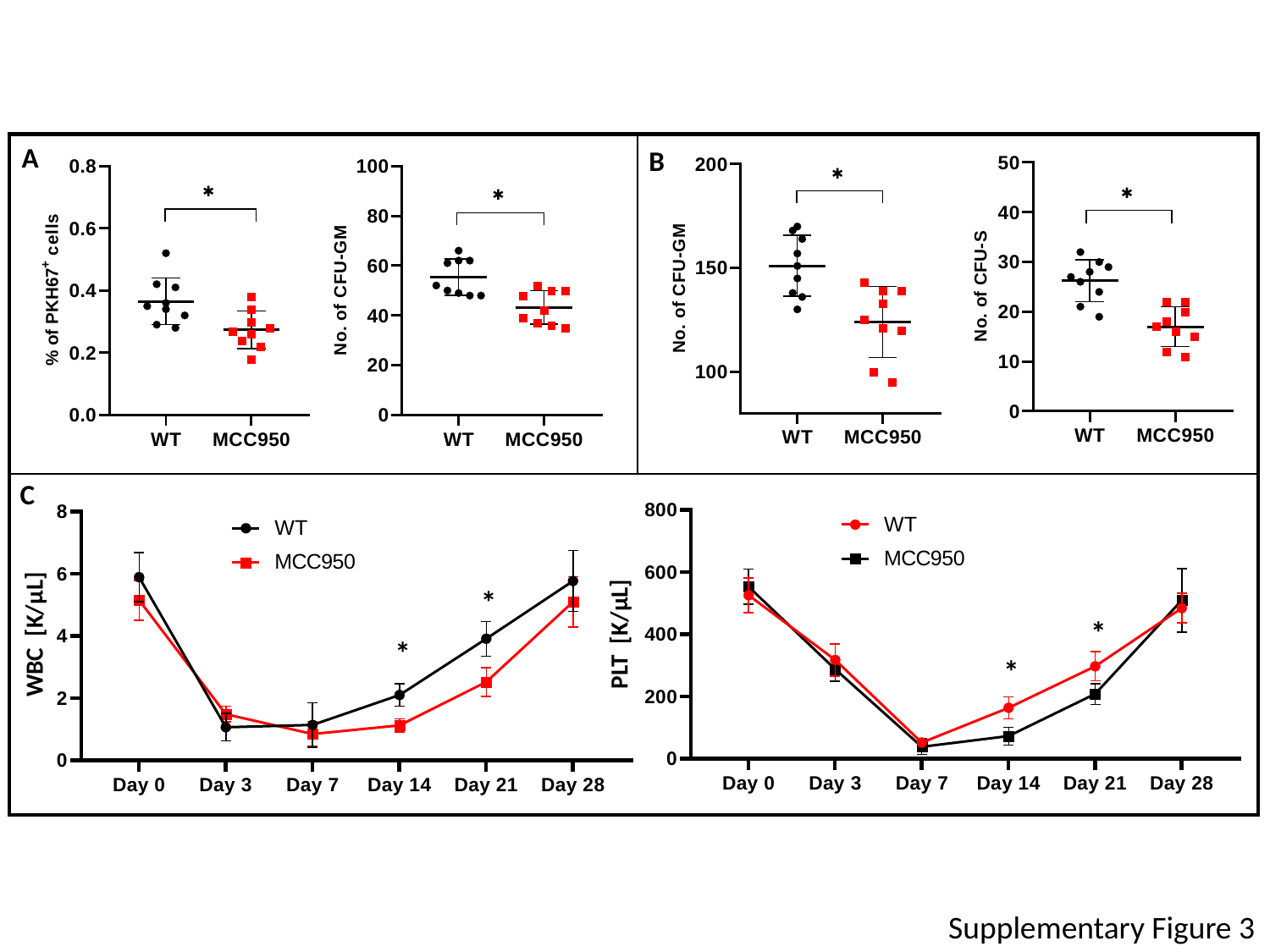

A
B
C
*
*
*
*
PLT [K/µL]
WBC [K/µL]
Supplementary Figure 3

Supplement: Supplementary file 3 — Defect in short- and long-term engraftment of HSPCs in WT mice treated with an Nlrp3-selective inhibitor. Panel A. Lethally irradiated WT mice (9 per group), untreated or treated with MCC950, were transplanted with bone marrow mononuclear cells (BMMNCs) from WT mice that had previously been labeled with a PKH67 cell linker. Twenty-four hours after transplantation, femoral BMMNCs were harvested, the number of PKH67+ cells evaluated by FACS, and the CFU-GM clonogenic progenitors enumerated in an in vitro colony assay. Panel B. Lethally irradiated WT mice (9 per group), untreated or treated with MCC950, were transplanted with BMMNCs from WT mice, and 12 days after transplantation femoral BMMNCs were harvested and plated to count the number of CFU-GM colonies and the spleens removed for counting the number of CFU-S colonies. No colonies were formed in lethally irradiated, untransplanted mice (irradiation control). *p < 0.05. Panel C. Lethally irradiated mice (9 per group), untreated or treated with MCC950, were transplanted with BMMNCs from WT mice. White blood cells (left) and platelets (right) were counted at intervals (at 0, 3, 7, 14, 21, and 28 days after transplantation). *p < 0.05. (PPTX 80 kb) [file 12015_2020_10005_MOESM3_ESM.pptx]
