# Supplementary material for: Nlrp3 Inflammasome Signaling Regulates the Homing and Engraftment of Hematopoietic Stem Cells (HSPCs) by Enhancing Incorporation of CXCR4 Receptor into Membrane Lipid Rafts
Source: Stem Cell Rev Rep. 2020 Jul 13;16(5):954–67. doi: 10.1007/s12015-020-10005-w (PMC7456406; doi:10.1007/s12015-020-10005-w)
Supplement: Supplementary file 4 — A reduced number of HSPCs in the BM of Nlrp3-KO mice. The number of SKL cells in the BM of Nlrp3-KO mice compared with WT control animals was evaluated by FACS and by the number of CFU-GM and BFU-E clonogenic progenitors in in vitro methylcellulose cultures. Results are combined from two independent experiments (4 mice per group per repeat). (PPTX 51 kb) [file 12015_2020_10005_MOESM4_ESM.pptx]

## Slide 1
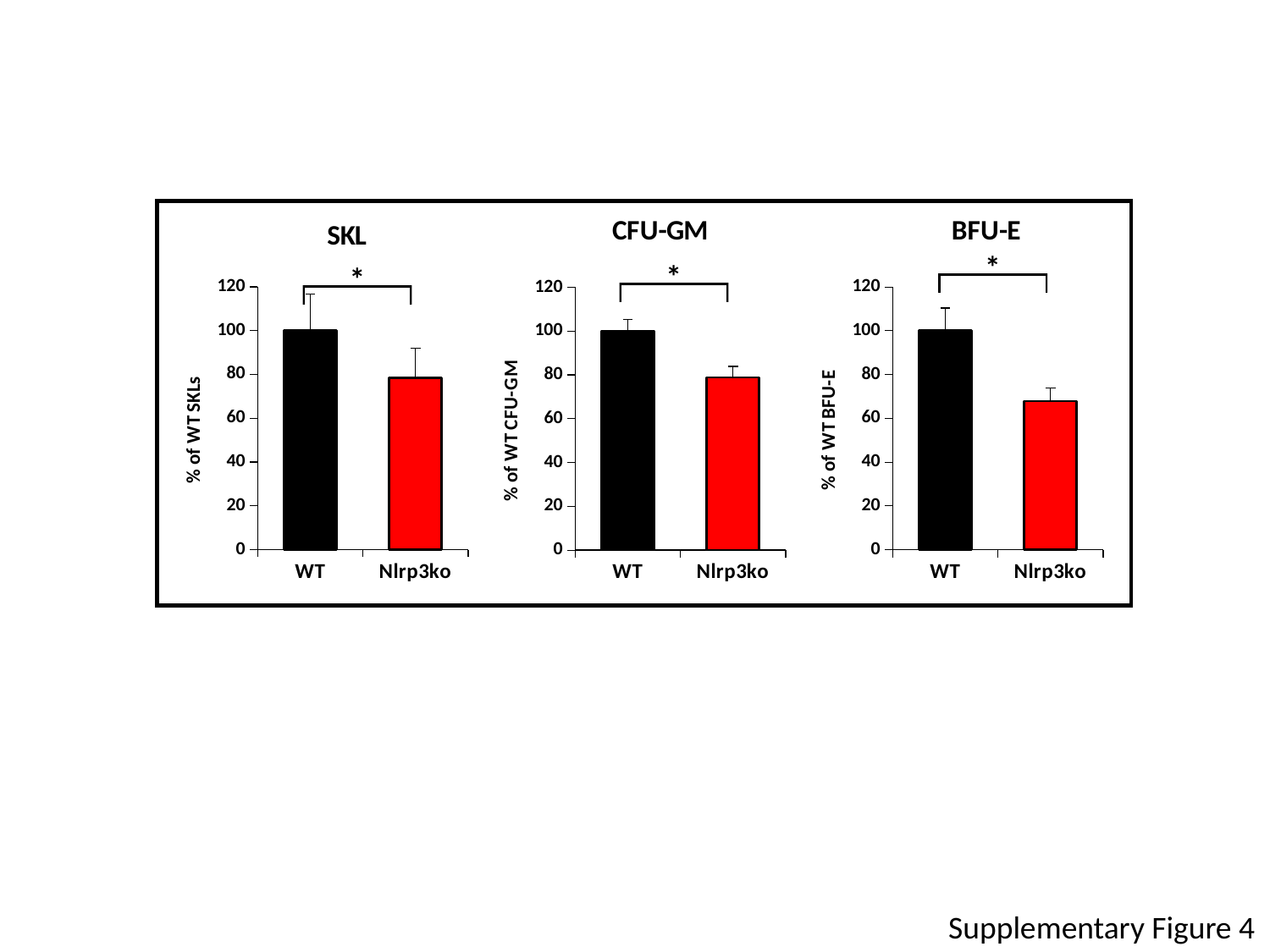

### Chart: SKL
| Category | |
|---|---|
| WT | 100.0 |
| Nlrp3ko | 78.4734494036349 |
### Chart: BFU-E
| Category | |
|---|---|
| WT | 100.0 |
| Nlrp3ko | 67.88083623693379 |
### Chart: CFU-GM
| Category | |
|---|---|
| WT | 100.0 |
| Nlrp3ko | 78.77023477701545 |*
*
*
Supplementary Figure 4
